# The Round-Up

**Published:** 1886-06

**Authors:** 


### THE ROUND-UP.

With this number closes Volume One. "Here ends the first lesson" as the good minister says; and we trust it has been a lesson by which our readers have been benefitted. This finishes, too, our first round trip on that "journalistic sea" of which we said so many pretty things when we "launched" the "New Texas." We have rounded to, and have made the landing, after a fair voyage and a quick run, encountering naught on the way but favoring breezes, and helping hands. We step ashore and make our obeisance gracefully, and tender our most grateful acknowledgements to our *compagnon's du voyage* who have not only borne us delightful company but who have, by their assistance, enabled us to make the most successful trip on record. To drop the stilts however, and come down to hard facts; this number closes a year of journalism of unprecedented success; the result has been most gratifying and encouraging. The outlook for the second round compared to that of the first, is as sunshine to "midnight, e'en in the zenith of her dark domain." We have had the satisfaction of seeing contributions to the pages of the JOURNAL, by men who, perhaps, had never before written a line for publication, copied and commented on in favorable terms, not only throughout the Union, but even in Europe.

Under the stimulus of repeated and impressive exhortation, organization has boomed, a number of counties having wheeled into line and gone to work; while over one hundred new members have come into the parent fold. Look at our twenty odd pages of the cream of medical advertising, that feature of journalism which delights the publishers' heart and makes glad ye printer; while our subscription list has swollen so rapidly that we are obliged to increase our monthly issue 25 per cent. From and after July twelve hundred and fifty copies will be issued; and the size of the JOURNAL will be increased to 76 pages, which will give our readers fifty-six to sixty pages of choice reading matter; and encouraged as we are, we will begin Vol. 2 with stout hearts and high hopes, and will endeavor to infuse into it all the vim, vigor and vitality engendered in our hopeful nature by the unprecedented and overwhelming favor with which Vol. 1 has been received. We want every physician in Texas to read the JOURNAL. It is edited and published in Texas by a Texan (by adoption), for Texans, and in the interest of the Texas State and County associations; and every physician who has the welfare of the profession at heart, and who desires progress, purification and elevation in Texas medicine is earnestly invited to co-operate in the endeavor to put Texas ahead in medical journalism as she is in agriculture and education, and all the elements that go to make up a great and glorious state. "*All aboard!*"

